# Can circulating tumor DNA be used for direct and early stage cancer detection?

**DOI:** 10.12688/f1000research.13440.1

**Published:** 2017-12-13

**Authors:** Eleftherios P Diamandis, Clare Fiala

**Affiliations:** 1 Department of Clinical Biochemistry, University Health Network, Toronto, Ontario, Canada; 2 Department of Laboratory Medicine and Pathobiology, University of Toronto, Toronto, Ontario, Canada; 3 Department of Pathology and Laboratory Medicine, Mount Sinai Hospital, Toronto, Ontario, Canada

**Keywords:** Circulating tumor, Cancer diagnosis, Cancer detection, Tumor measurement, Colorectal, breast, lung, ovarian cancer data

## Abstract

In the August 16th issue of Science Translational Medicine, Phallen et al propose a method for early cancer diagnosis by using circulating tumor DNA (1). One major advance of this paper includes optimized sequencing of cell-free/circulating tumor DNA (ctDNA) without knowledge of tumor mutations. Evaluation of 200 patients with colorectal, breast, lung and ovarian cancer revealed mutations in ctDNA in approx. 60-70% of all patients, including stage 1 and stage 2 disease. If this data can be reproduced in asymptomatic individuals, they will likely have a major impact on early cancer detection and patient outcomes. In this commentary, we examine the feasibility of this approach for detecting small, asymptomatic tumors, based on previously published empirical data.

## Correspondence

Phallen *et al.* recently proposed a method for early cancer diagnosis by using circulating tumor DNA ^1^. One major advance of this paper includes optimized sequencing of cell-free/circulating tumor DNA (ctDNA) without knowledge of tumor mutations. Evaluation of 200 patients with colorectal, breast, lung and ovarian cancer revealed mutations in ctDNA in approximately 60–70% of all patients, including stage 1 and stage 2 disease. If this data can be reproduced in asymptomatic individuals, they will likely have a major impact on early cancer detection and patient outcomes. In this correspondence, we examine if this approach is effective in detecting small, asymptomatic tumors, based on previously published empirical data.

An important question is the desirable size of a tumor to be detected with such methods. For this discussion, we will use data mostly from breast cancer, for which there is ample experience from screening programs. Some organizations set a tumor diameter of 1 mm as the goal of early detection ^2^ since tumors of this size are localized, less complex and likely to be cured by radical resection. The literature suggests that the chances of progression of these small, 1 mm diameter tumors, is less than 1% ^3^. However, such small tumors could be found in many asymptomatic individuals and this may lead to over-diagnosis and over-treatment ^3^. Consequently, we also include larger tumors in this discussion. It could be argued that 5 mm in diameter is an optimal tumor size for early and curable cancer detection. At 5 mm diameter, the chances of this tumor progressing are small (around 6%) ^3^, and currently, these small lesions are only detectable by mammography (or other imaging modalities) in only 26% of cases ^4^. We also suggest that detection of a 10 mm diameter tumor may not be an advance in breast cancer screening, since at this size, the chances of progression increase considerably (to about 50%), and most of these tumors (> 90%), are currently detected by mammographic screening.

We further assume that the circulating free DNA concentration (cfDNA) in individuals without cancer, or very small cancers (ctDNA), is about 5 ng/mL on average ^5^, which is equivalent to about 6,000 whole haploid genomes per 4 mL of plasma (10 mL blood draw). For this discussion we also use well-established measurements of tumor volume and cellularity. A tumor of approximately 12.5 mm in diameter, weights approx. 1 gram, has a volume of 1 cm ^3^ and contains approx. 100 million to 1 billion cells ^6^. According to recent estimates of ctDNA and tumor volume, a 10 gram tumor has a volume of 10 cm ^3 7^. For such tumors, the average percent fraction of mutant DNA has been reported to be 0.1 %, or 1 mutant DNA molecule per 1,000 non-mutated DNA molecules in the circulation ^7^.

Based on these published assumptions, we constructed Table 1.

**Table 1.** Tumor measurements and chances of progression or detection by mammographic screening (assuming spherical tumor).

Tumor diameter, mm	Tumor weight, mg	Tumor Volume, mL(cm ^3^)	Number of cells	%Fraction of mutant DNA	Number of genomes per 10 mL blood/4 mL plasma	Chance of progression ^3^	Mammographic screen sensitivity ^4^
27	**10,000**	**10 ^1^**	**1,000,000,000**	**1:1,000**	6		
12.5	**1,000**	**1 ^2^**	**100,000,000**	1:10,000	0.6		
10	500	0.5	50,000,000	1:20,000	0.3	50%	91%
6	125	0.12	12,000,000	1:80,000	<0.1		
5	62	0.06	6,000,000	1:160,000	<0.1	6%	26%
3	16	0.015	1,000,000	1:640,000	<0.1		
2	4	0.0035	400,000	1:2,600,000	<0.1		
1.1	1	0.0008	100,000	1:10,000,000	<0.1	0.05%	

1. As reported in Ref 7; bold font indicates experimental data. Other data were calculated by extrapolation 2. As reported in Ref 6 3. As reported in Ref 3 4. As reported in Ref. 4

Table 1 shows the minimum tumor measurements required for 4 mL of plasma to contain at least 1 mutated genome. The percent fraction of mutant DNA is approximately 1 in 10,000 (0.01%) which corresponds to a tumor volume of 1 cm ^3^ or 12.5 mm in diameter (assuming a spherical nodule). Most, if not all, of these tumors are currently detectable by mammographic screening or other imaging modalities, which have an approximate limit of detection of about 4 mm diameter ^8^. Table 1 also shows that when the tumor diameter drops below 10 mm, the chances of this method working are minimal, since there will be not enough tumor DNA (at least one copy) in 4 mL of plasma to make detection possible. Consequently, Table 1 predicts that 5 mm diameter tumors will not be detected due to this sampling error. These estimates are corroborated by the data presented by Phallen *et al.* ^1^. They used tumors of varying sizes, stages and types, but universally, the mutant fraction of ctDNA is never less than 0.01%, the approximate threshold for 1 genome copy in 4 mL of plasma. We do not know if the patients included in the study have been preselected to have a specific cut-off of percent mutant DNA in the circulation. If this is the case, the data will be biased towards detection of tumors which are rather large (> 10 mm in diameter and have a mutant fraction of > 0.01%). In fact, >95% of their cases have mutant fraction > 0.1% (Figure 5 from Phallen *et al.* ^1^ which suggests tumor sizes of > 27 mm ( Table 1). We anticipate that in a real case scenario, small and asymptomatic tumors will likely yield percent mutant DNA fraction of much less than 0.1%, thus making detection of these tumors highly unlikely, due to sampling error.

The sensitivity of the method proposed by Phallen *et al.* is further compromised by the number of genes tested for mutations (currently 58 genes). The predicted maximum sensitivity is about 80%, assuming best case scenario of 1 mutation detected to signify cancer. If we assume that the overall positivity of this test is 50%, then, the overall sensitivity will likely be less than 40%. This would make screening of asymptomatic individuals less efficient, since the majority of patients will receive incorrect screening results (false negatives) due to either lack of the targeted mutations or sampling error.

The specificity of this method is claimed to be very high due to detection of only driver mutations. However, as shown in Phallen *et al.*’s Supplementary Figure S3 ^1^, the gene with the highest sensitivity for cancer detection of all of the 4 cancer types is p53. However, there are multiple reports demonstrating mutations of p53 in apparently healthy individuals, ranging from 0.2% to 11% ^8– 11^. If we assume an overall specificity of 99% and an overall sensitivity of 40% with this test, and a prevalence of cancer around 1%, then in 1,000 screened individuals, there will be 4 true positive, 6 false negative, 10 false positive and 992 true negative results. The positive predictive value (PPV) will be only 29%. At 98% specificity, the PPV further drops to 20%. Not only would more than half the patients will receive incorrect (false negative) results but about 72–80% of tested positive patients would need to undergo additional diagnostic procedures to rule-in or rule-out cancer. Additional specificity concerns may include technical noise and somatic mutations stemming from other biological phenomena. Also, in this paper, no matched DNA was employed to differentiate somatic from germline mutations, another important limitation ^12^.

Our analysis shows that measurement of ctDNA for early cancer diagnosis is problematic, not only due to the limited capability of deep whole genome sequencing to reveal mutations, but because the amount of circulating tumor DNA retrieved from a 10mL blood draw will be extremely small, or even nonexistent, thus making efficient diagnosis of cancer unlikely. Our analyses generally show that when the tumor diameter drops below 10 mm, not a single mutant DNA copy will be retrieved. We do not exclude the possibility of future additional technical improvements, so that mutant DNA could somehow be extracted from the whole circulation and subjected to such deep sequencing, to increase sensitivity, provided that the specificity of the test would be preserved to close to 100%. Otherwise, under a screening scenario, these methods are unlikely to work. Our analyses also show that a 1 mm tumor is likely associated with 1 copy of ctDNA in the entire circulation, further underlining the difficulty in detection. The possibility exists that early cancer detection may be possible in specific clinical contexts or specific tumor types using this method. Also, it may be possible to combine protein and molecular markers to increase sensitivity, as shown already for pancreatic cancer ^13^.

We hope that our analyses will help to further understand the opportunities and limitations of ctDNA as a cancer biomarker, especially for early cancer detection.
