# Supplementary material for: Can circulating tumor DNA be used for direct and early stage cancer detection?
Source: F1000Res. 2017 Dec 13;6:2129. [Version 1] doi: 10.12688/f1000research.13440.1 (PMC5754742; doi:10.12688/f1000research.13440.1)
Supplement: Peer review history

# Peer review: Referee response for version 1

**DOI:** 10.5256/f1000research.14593.r29016

**Published:** 2018-01-04

**Authors:** Catherine Alix-Panabières

**Affiliations:** 1 Laboratory of Rare Circulating Human Cells (LCCRH), Department of Pathology and Onco-Biology, University Medical Center of Montpellier, Montpellier, France

This commentary, very well written by the expert Eleftherios P Diamandis and his colleague Clare Fiala, is based on the last article of Phallen *et al.* who described a method for early cancer diagnosis by using circulating tumor DNA (ctDNA) sequenced without knowledge of tumor mutations.

 In this commentary, the authors examined step by step the feasibility of this approach for detecting small, asymptomatic tumors, based on previously published empirical data. They nicely showed that measurement of ctDNA for early cancer diagnosis is problematic, not only due to the limited capability of deep whole genome sequencing to reveal mutations, but because the amount of circulating tumor DNA retrieved from a 10mL blood draw will be extremely small, or even nonexistent, thus making efficient diagnosis of cancer unlikely.

 This commentary, very well documented, is important in the liquid biopsy field and needs to be indexed to understand the current opportunities and limitations of ctDNA as a biomarker for early detection of cancer.

I have read this submission. I believe that I have an appropriate level of expertise to confirm that it is of an acceptable scientific standard.

# Peer review: Referee response for version 1

**DOI:** 10.5256/f1000research.14593.r29018

**Published:** 2017-12-14

**Authors:** Nicholas J. Wald, Robert W Old

**Affiliations:** 1 Wolfson Institute of Preventive Medicine, Barts and The London School of Medicine and Dentistry, Queen Mary University of London, London, UK

This paper is a commentary on a study by Phallen *et al*. who developed and applied advanced DNA analysis techniques for circulating tumor DNA from patients with untreated breast, colorectal and ovarian cancer. They were able to detect cancer in about 60-70% of patients with Stage I or Stage II disease. Diamandis and Fiala’s Commentary examines the feasibility of applying these techniques to small tumors that are not reliably detectable by other means. They extrapolate original data from Phallen *et al*., making the assumption that the number of tumor-derived genomes in the circulation is proportional to tumor volume. Diamandis and Fiala reasonably conclude that tumors smaller than about 10mm in diameter result in less than one tumor-derived genome per 10ml blood draw and are therefore, in principle, undetectable in practice. This logic is probably conservatively based because small, low grade tumors are likely to produce proportionally less ctDNA than high grade tumors. This Commentary is a useful contribution to the literature.

We have read this submission. We believe that we have an appropriate level of expertise to confirm that it is of an acceptable scientific standard.
